# Temperature-regulated defective MIL-100(Fe) for clove essential oil loading as an effective natural preservative for peaches

**DOI:** 10.3389/fnut.2026.1755325

**Published:** 2026-04-16

**Authors:** Naiding Wang, Jun Zhang, Rongpeng Li, Yanqing Duan, Ruizhi Zhu, Lei Yang, Haotian Yang, Yuandong Li, Juxing Jiang, Yanqun Xu, Zhigang Tai

**Affiliations:** 1 Yunnan Tobacco Industrial Hi-tech Material Co., Ltd., Kunming, China; 2 Faculty of Science and Life Technology, Kunming University of Science and Technology, Kunming, China; 3 R&D Central of China Tobacco Yunnan Industrial Co., Ltd., Kunming, China

**Keywords:** antibacterial activity, clove essential oil, defective MIL-100(Fe)-x, peach preservation, sustained release

## Abstract

The perishability of fruits and vegetables poses significant challenges for their transportation and storage. Clove essential oil (CEO) is a natural preservative, but its high volatility and instability limit its practical application. In this study, a series of novel defective MIL-100(Fe)-x (D-MIL-100(Fe)-x, where x represents the molar ratio relative to iron: 1, 5, 10, or 15) were synthesized using trifluoroacetic acid (TFA) as a modulator and subsequently loaded with CEO to enhance stability and enable sustained release for peach preservation. Among these, D-MIL-100(Fe)-1 exhibited the highest CEO loading capacity of 610.6 mg/g, 1.45 times greater than that of conventional MIL-100(Fe). The composite demonstrated significant sustained-release behavior, releasing 42.3% of CEO over 16 days at 25 °C. D-MIL-100(Fe)-1/CEO showed a preservative effect comparable to that of the refrigerated group, offering enhanced versatility, convenience, and cost-effectiveness. This study provides a novel defective MIL-100(Fe)-x for effective essential oil loading, providing a safe and biocompatible method for preserving fruits and vegetables.

## Graphical abstract

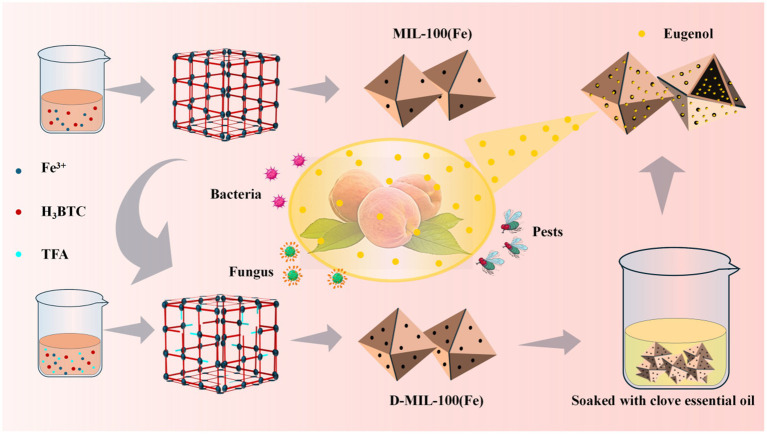

## 1 Introduction

Fruits and vegetables are integral components of the human diet because they provide essential nutrients that support overall health. However, their high perishability poses significant challenges during transportation and storage (1). Currently, preservation methods often rely on chemical fungicides such as NaClO, HOCl, and ClO_2_ (2). The low cost of these agents commonly leads to their excessive use, resulting in problems such as pathogen resistance and the accumulation of chemical residues (3). Therefore, developing eco-friendly and highly effective preservatives to replace traditional chemical agents has become an urgent priority for sustainable food preservation (4).

Essential oils (EOs), volatile compounds naturally extracted from plants such as *Syzygium aromaticum*, exhibit several biological activities, including anti-inflammatory, antibacterial, antifungal, and antiviral effects, that inhibit microbial growth and thereby enhance preservation (5–8). Clove essential oil (CEO), predominantly composed of eugenol, has been widely recognized as an effective natural food preservative due to its potent antibacterial and antioxidant properties, coupled with its high safety profile (9, 10). However, CEO is highly volatile, chemically unstable, and susceptible to oxidation, which limits its effectiveness in preservation applications (11). Recently, efficient delivery systems, including encapsulation and spice precursor technologies, have been developed to achieve controlled release of volatile compounds for preservation purposes (10). Encapsulation is a practical approach in which various EOs are physically entrapped within matrices or capsules without requiring complex processing, as previously reported by previous research (12–14). The release mechanism primarily involves diffusion, which is influenced by the physicochemical properties of the EO, as well as the shell thickness and structural permeability of the carrier materials (15). However, certain encapsulating materials may induce the instantaneous release of active compounds upon exposure to external stimuli. Moreover, EOs encapsulated in emulsions may partially lose their intrinsic characteristics (16). Therefore, developing novel encapsulation materials remains essential for expanding the application of EOs in preservation.

In recent years, metal–organic frameworks (MOFs) have attracted considerable attention as carriers for active compounds due to their periodic porous structures, high specific surface area, and tunable porosity (17). Studies have shown that MOFs can effectively store EOs and regulate their sustained release (18). However, EOs often contain molecules of varying sizes. Conventional MOFs, with their highly ordered crystalline structures and uniform pore-size distributions, readily adsorb small molecules but hinder the diffusion of larger ones, thereby limiting their effectiveness in sustained-release applications (19). Defect engineering, a modification strategy that introduces controlled defects into MOFs, can regulate their specific surface area, pore structure, and porosity, leading to the formation of hierarchical pores and increasing the availability of active sites capable of accommodating molecules of different sizes (20). For instance, defect-engineered UiO-66, a liganded MOF, can increase its pore size from 0.8 to 1.2 nm and improve total porosity by 10–30%, thereby enhancing adsorption capacity and selectivity for a variety of molecules (21). These characteristics indicate that defective MOFs, with their large surface areas, high porosity, and design flexibility, can optimize the adsorption and release performance of EOs with complex compositions, ultimately improving their application in food preservation.

MIL-100(Fe) is a type of MOF formed through the coordination of Fe^3+^ with trimellitic acid. It is biocompatible under physiological conditions and can be metabolized by the body. Owing to its large specific surface area, high porosity, and tunable pore architecture, MIL-100(Fe) has been widely investigated as a drug carrier in delivery systems (22). These intrinsic properties also suggest that MIL-100(Fe) is a promising carrier for EOs in preservation applications. Defective-MIL-100(Fe) regulated by benzoic acid, retains the characteristics of high specific surface area and porosity while offering improved pore structures, enabling prolonged release of active compounds and improved preservation performance (23). In addition, modulating defect density using TFA has emerged as an effective strategy to increase the surface area, enhance porosity, and tailor the pore architecture of MOF composite, such as MOF-808, UiO-66(Zr) and MOF-545 (24–26). However, to our knowledge, TFA-regulated defective-MIL-100(Fe) has not been reported. Given that TFA can act as a hydrogen bond acceptor, it is expected to enhance the affinity of defective-MIL-100(Fe) for eugenol in CEO. Consequently, the potential of this material for CEO adsorption and sustained release, particularly its application in food preservation, warrants further systematic investigation.

In this study, TFA-modulated defective MIL-100(Fe) was synthesized for the first time. Its structure was characterized using scanning electron microscopy (SEM), Fourier transform infrared spectroscopy (FTIR), X-ray diffraction (XRD), and X-ray photoelectron spectroscopy (XPS). Subsequently, the CEO was extracted from *Syzygium aromaticum* and loaded into the defective MIL-100(Fe) to obtain D-MIL-100(Fe)/CEO, and it’s structure was further verified using FTIR, TGA (thermogravimetric analysis), and XRD. Moreover, the release behavior of D-MIL-100(Fe)/CEO was investigated at 25 °C, 35 °C, and 45 °C. Finally, the preservative efficacy of D-MIL-100(Fe)/CEO was evaluated using peaches as the model system. Overall, this study aimed to develop a simple, efficient, and biocompatible D-MIL-100(Fe) capable of achieving controlled release of CEO, thereby providing an innovative material for food preservation.

## 2 Materials and methods

### 2.1 Reagents and materials

*Syzygium aromaticum* was purchased from a Chinese herbal medicine market in Kunming, China, and subsequently dried, ground, and sieved through a 40-mesh screen for extraction. The reagents used in this study included iron powder (Fe, 98%), trimellitic acid (H₃BTC, 98%), hydrofluoric acid (HF, 49%), nitric acid (HNO₃, 65%), and trifluoroacetic acid (TFA, 99%), all purchased from Shandong Keyuan Bio-Chemical Co., Ltd. Anhydrous ethanol (>99.7%) and hexane (98%) were purchased from Tianjin Fengchuan Chemical Reagent Technology Co., Ltd. All aqueous solutions were prepared using deionized water. 2,2-Diphenyl-1-picrylhydrazyl (DPPH) and ampicillin were purchased from Sinopac Group Co., Ltd. *Staphylococcus aureus*, *Escherichia coli*, and *Bacillus subtilis* were obtained from the Agricultural Culture Collection of China (ACCC) (Beijing, China).

### 2.2 Preparation of MIL-100(Fe) and defective MIL-100(Fe)

#### 2.2.1 Preparation of MIL-100(Fe)

MIL-100(Fe) was synthesized following a previously described method (27). Briefly, 0.82 g of Fe and 2.06 g of H₃BTC were dissolved in 80 mL of deionized water. Subsequently, 1.2 mL of HNO₃ (65%) and 0.5 mL of HF (49%) were added to the solution. The mixture was vigorously stirred for 1 h and transferred to a high-pressure reactor. The reaction was carried out at 150 °C for 24 h. After cooling to room temperature, the resulting precipitate was collected by centrifugation and washed with deionized water at 80 °C to remove residual reactants. The final product was dried in a vacuum oven at 60 °C for 12 h.

#### 2.2.2 Preparation of D-MIL-100(Fe) -x

The synthesis of D-MIL-100(Fe)-x was essentially identical to that of MIL-100(Fe), with the addition of TFA as a modulator. Briefly, TFA was added to the reaction mixture at different molar ratios relative to iron, expressed as 1:x (where x represents 1, 5, 10, or 15). The Fe-to-TFA molar ratio was precisely controlled to adjust the defect concentration during synthesis. The resulting materials were designated as D-MIL-100(Fe)-1, D-MIL-100(Fe)-5, D-MIL-100(Fe)-10, and D-MIL-100(Fe)-15, respectively.

### 2.3 Optimization of CEO extraction

CEO was extracted from *Syzygium aromaticum* using steam distillation, following the method described by Guan et al. (28). To maximize the CEO yield, a single-factor experimental design was employed to determine the optimal extraction parameters. As reported by Hu et al. (29), the initial extraction conditions were as follows: the NaCl concentration was varied from 1 and 9%, the liquid to solid ratio was adjusted to 5, 10, 15, 20, or 25 mL/g, and the extraction time was set to 2, 2.5, 3, 3.5, or 4 h. The optimal extraction conditions were identified based on the maximum yield of CEO.

### 2.4 Preparation of D-MIL-100(Fe)-x/CEO

D-MIL-100(Fe) -x/CEO was prepared following the method described by Zhang et al. (30), with slight modifications. Initially, 50 mg of D-MIL-100(Fe)-x was dried in a vacuum oven at 80 °C for 30 min. Subsequently, 2 mg of CEO was mixed with the D-MIL-100(Fe)-x, and the mixture was shaken for 24 h to facilitate CEO loading. Resulting D-MIL-100(Fe)-x/CEO was then centrifuged at 8,000 rpm for 10 min. The precipitate was washed with 5 mL of hexane to remove CEO adsorbed on the surface and dried in a vacuum oven at 40 °C for 15 min to eliminate residual solvent.

### 2.5 Characterization

#### 2.5.1 Morphology

Surface morphology of MIL-100(Fe) and D-MIL-100(Fe)-x was examined using an SEM (Sigma 300, Carl Zeiss AG, Germany). The samples were coated with a thin layer of gold and observed under an accelerating voltage of 5 kV.

#### 2.5.2 XPS analysis

To examine the incorporation of TFA into the framework of D-MIL-100(Fe)-x, XPS analysis of MIL-100(Fe), and D-MIL-100(Fe)-1 was performed using an XPS instrument (Nexsa G2, Thermo Fisher Scientific, USA).

#### 2.5.3 Nitrogen adsorption–desorption

The surface area and porosity of MIL-100(Fe) and D-MIL-100(Fe)-x were determined using the Brunauer–Emmett–Teller (BET) method with a gas adsorption analyzer (ASAP 2460, Micrometrics, USA). Before analysis, the samples were soaked in CH2Cl2 to remove residual solvents from the pores.

#### 2.5.4 Crystal structure

The crystal structure of MIL-100(Fe), D-MIL-100(Fe)-x, and D-MIL-100(Fe)-x/CEO were analyzed using an XRD (MiniFlex 600, Rigaku, Japan). Scans were performed with a step size of 0.02° over a 2θ range of 5° to 40°.

#### 2.5.5 FTIR spectroscopy

Chemical bonds and functional groups in CEO, D-MIL-100(Fe)-x, and D-MIL-100(Fe)-x/CEO were analyzed using an FTIR spectrophotometer (iS20, Thermo Fisher Scientific, USA). The samples were mixed with KBr and pressed into pellets for measurement. Spectra were recorded over the range of 400–4,000 cm^−1^ with a resolution of 4 cm^−1^.

#### 2.5.6 Thermal properties

The thermal stability of MIL-100(Fe), D-MIL-100(Fe)-1, and MIL-100(Fe)/CEO was evaluated using thermogravimetric analysis (TGA/DSC 3+, Mettler Toledo, Switzerland) over a temperature range of 30–800 °C at a heating rate of 20 °C/min.

### 2.6 Loading capacity of CEO

The loading capacity (LC,%) of D-MIL-100(Fe)-x/CEO was determined following a previously described method (25), with slight modifications. Briefly, 10 mg of D-MIL-100(Fe)-x/CEO was dispersed in 10 mL of ethanol and sonicated for 5 min to extract the CEO. The resulting solution was diluted tenfold and analyzed at 282 nm using a UV–visible spectrophotometer (UV-1800, Shimadzu, Japan). As shown in Supplementary Figure S1, the concentration of CEO was calculated using an external standard method (A = 0.0230 + 0.0181C, R2 = 0.9987). LC(%) was calculated using Equation 1:


$$\;\;\text{Loading capacity}(\mathrm{LC}\%)=\frac{m}{m_{0}}\times 100$$ (1)

Where m represents the amount of CEO encapsulated in the sample and m0 represents the total amount of carrier.

### 2.7 *In vitro* release

The *in vitro* release of D-MIL-100(Fe)/CEO was assessed following the method described by Thing et al. (31). Briefly, D-MIL-100(Fe) /CEO (150 mg) were equilibrated in a thermostated chamber at 25, 35, or 45 °C. At predetermined intervals, 5 mg of the sample was collected, dispersed in 15 mL of ethanol, and sonicated at 80% amplitude for 5 min. The mixture was then vigorously agitated for 30 min to extract CEO, and the resulting solution was separated by centrifugation at 5,000 r/min for 10 min. The precipitate was washed with ethanol, and the supernatant extraction was repeated three times. CEO content was quantified by UV–visible spectrophotometry (UV-1800, Shimadzu, Japan), and cumulative release (CR, %) was calculated using Equation 2:


$$\text{Cumulative release}(\mathrm{CR}\%)=\frac{m}{m_{0}}\times 100$$ (2)

Where *m* represents the amount of CEO released from D-MIL-100(Fe)-x/CEO, and *m_0_* represents the initial amount of CEO in D-MIL-100(Fe)/CEO.

To determine the kinetic model that best described the release pattern of CEO from D-MIL-100(Fe)/CEO, the cumulative release data were fitted to three models: Zero-order, First-order, and the Ritger–Peppas model (Equations 3–5).

*Zero-order:*


$$\;q=k_{1}t+c_{1}$$ (3)

*First-order:*


$$q=1-e^{-k_{2}t}$$ (4)

*Ritger–Peppas*:


$$\;q=k_{3}t^{n}$$ (5)

Where t represents the release time; k_1_, k_2_, and k_3_ represent the rate coefficients for the Zero-order, First-order, and Ritger–Peppas, respectively. And n represents the release exponent, which provides insight into the underlying transport mechanism. Model selection was based on the coefficient of determination (*R^2^*), with the model yielding the highest *R^2^* considered the most accurate descriptor of CEO release from D-MIL-100(Fe)/CEO.

### 2.8 Antioxidant and antibacterial activity assay

#### 2.8.1 Antioxidant assay

The antioxidant capacity of the samples was evaluated using the DPPH free radical scavenging assay. The analysis was performed following the method described by Tai et al. (32), with slight modifications. Briefly, 20 mg of D-MIL-100(Fe)/CEO was dispersed in 5 mL of methanol and equilibrated at 25 °C for 12 h. Then, 100 μL of the sample extract was added to 4.0 mL of DPPH solution (0.1 mM). After incubation in the dark at room temperature for 30 min, the absorbance was measured at 517 nm. Methanol (100 μL) mixed with 4.0 mL of DPPH solution (0.1 mM) served as the blank control. The scavenging efficiency was calculated as the percentage inhibition using Equation 6.


$$\text{DPPH scavenging capacity}(\%)=\frac{A_{0}-A_{s}}{A_{0}}\times 100$$ (6)

Where *A_0_* and *A_s_* represent the absorbance of the blank control and the sample, respectively.

#### 2.8.2 Antibacterial assay

The antibacterial activity was evaluated using the agar disk diffusion method, following the procedure reported by Khaled-Khodja (33), with slight modifications. Samples were disinfected under UV light for 40 min, and all subsequent dilution and inoculation steps were performed on an open bench. Suspensions of *Staphylococcus aureus*, *Escherichia coli*, and *Bacillus subtilis* were adjusted to 1 × 10^6^ CFU/mL. Four wells, each 5.0 mm in diameter, were aseptically cut into the agar of each Petri dish. CEO and D-MIL-100(Fe)/CEO solutions were added to separate wells, and each well was overlaid with a drop of molten agar to prevent leakage. The plates were incubated at 37 °C for 24 h, and antibacterial activity was assessed by measuring the diameter of the inhibition zone. A plate without a sample served as the negative control, while ampicillin was used as the positive reference.

### 2.9 Preservation assay

The peach preservation experiment was conducted following the method described by Cui et al. (34), with slight modifications. Fresh peaches were harvested from Chenggong District, Yunnan Province, and held at 20 °C for 4 h after harvest. The peaches were randomly assigned to three groups: control, treatment, and cold-storage. The control and treatment groups were stored at 25 ± 1 °C with a relative humidity of 75 ± 5%. On the other hand, the cold-storage group was stored at 4 ± 0.5 °C and 90% relative humidity. Samples from all groups were stored in the dark for 10 days.

Peach quality was assessed in terms of appearance, hardness, titratable acidity (TA), soluble solids content (SSC), and total bacterial count (TBC) (35). The appearance was observed and photographed to document changes. Hardness was determined using a fruit hardness tester (HP-50, Bareiss, Germany) equipped with an 8 mm probe, with a penetration speed of 1 mm/s to a depth of 8 mm. For SSC analysis, peach samples were homogenized, filtered, and measured using a digital refractometer (J57-HE, Rudolph Research Analytical, USA). TA was determined by first incubating the homogenized peach samples in a water bath at 55 °C for 1 h. Subsequently, 5.0 g of the sample was mixed with 50 mL of water, and TA was measured using 0.05 mol/L NaOH using a pH meter (G20, Mettler Toledo, Switzerland). TA was calculated using Equation 7:


$$\mathrm{TA}\;(\text{mmol}/100\;g)=\frac{C\times V\;}{5}\times 100$$ (7)

Where C represents the concentration of NaOH (mol/L) and V represents the volume of NaOH (mL).

TBC was determined following the method previously reported by our group (35). Briefly, peach (10 g) was homogenized with 90 mL of sterile water for 30 min. An appropriate aliquot of the homogenate was spread onto plate count agar plates. After incubation at 37 °C for 48 h, TBC was calculated using Equation 8 and expressed as Log₁₀ CFU/g.


$$\mathrm{TBC}\;(\log_{10}\mathrm{CFU}/g)=\frac{N\times 90\;}{V}\times 10$$ (8)

Where N represents the number of colonies counted and V represents the volume of the homogenate sample plated.

### 2.10 Statistical analysis

All statistical analyses were performed using SPSS software (version 25, SPSS Inc., USA). Data were analyzed using one-way ANOVA followed by Duncan’s multiple-range test to assess significance, with *p* < 0.05 considered statistically significant. All experiments were conducted in triplicate, and results were presented as the mean ± standard deviation.

## 3 Results and discussion

### 3.1 Structural and morphology characterization

#### 3.1.1 Morphological analysis

**Figure 1:**
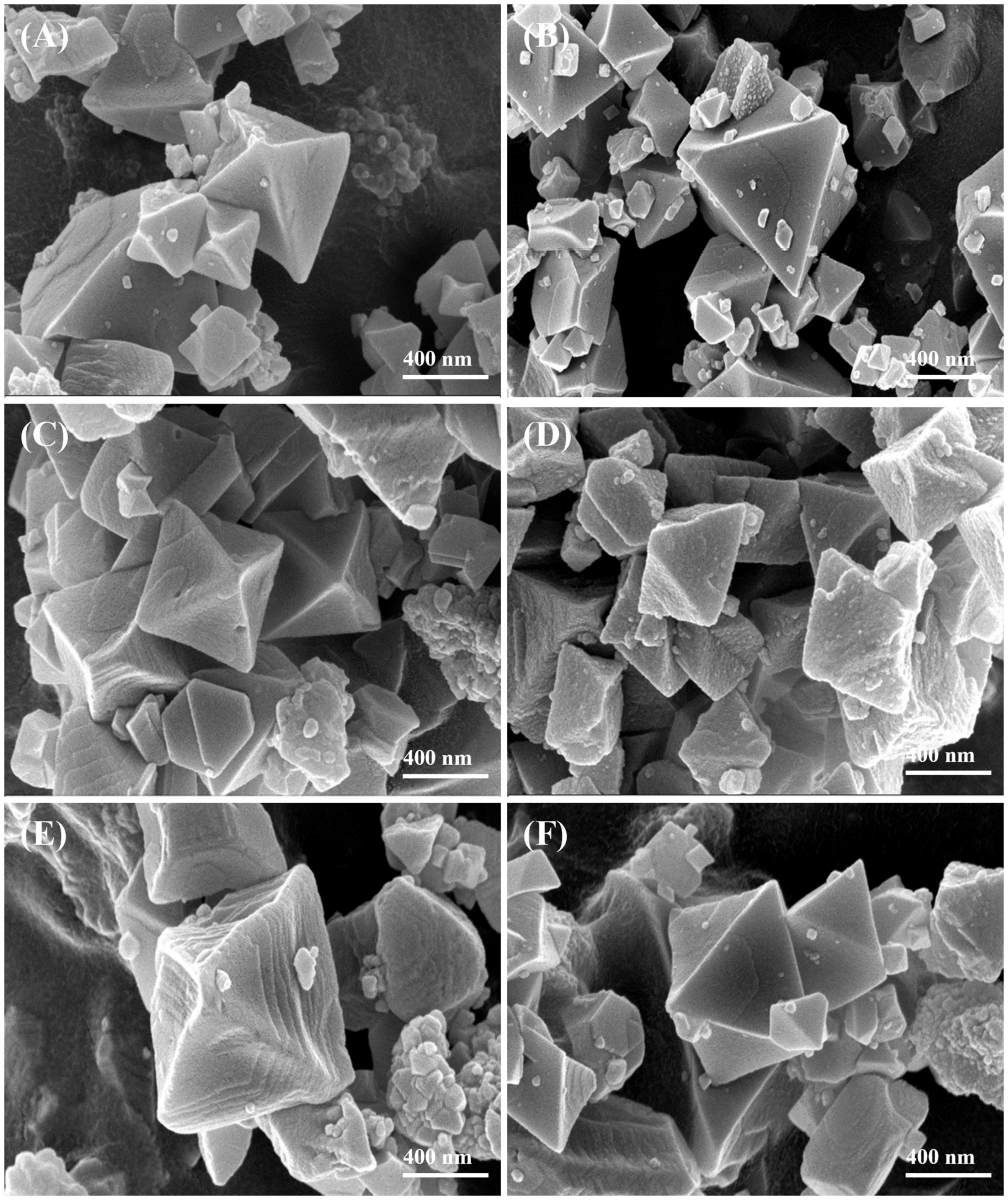
SEM images: **(A)** MIL-100(Fe); **(B)** D-MIL-100(Fe)-1; **(C)** D-MIL-100(Fe)-5; **(D)** D-MIL-100(Fe)-10; **(E)** D-MIL-100(Fe)-15; **(F)** D-MIL-100(Fe)-1/CEO.

As shown in Figure 1A, conventional MIL-100(Fe) synthesized without TFA displayed well-defined octahedral crystals, in agreement with previous reports (36). With the addition of low concentrations of TFA, the samples D-MIL-100(Fe)-1 and D-MIL-100(Fe)-5 exhibited more uniform octahedral crystals along with smoother surfaces (Figures 1B,C). As the TFA concentration further increased, the octahedral morphology was retained. However, the crystal surfaces became rougher and developed pronounced granular features (Figures 1D,E). These morphological changes are likely attributable to coordination interactions between TFA and Fe^3+^, which modulate the nucleation and growth processes of D-MIL-100(Fe)-x. Notably, after loading with CEO, D-MIL-100(Fe)-1/CEO showed no significant alteration in crystal shape (Figure 1F), indicating that CEO did not disrupt the structural integrity of D-MIL-100(Fe)-1. Similar observations have been reported in previous studies. Chen et al. (37) reported that loading curcumin into *γ*-CD-MOF exhibited a uniform structure, and Yu et al. (38) demonstrated that encapsulation of terpinene-4-ol did not induce observable morphological changes.

#### 3.1.2 XPS analysis

**Figure 2:**
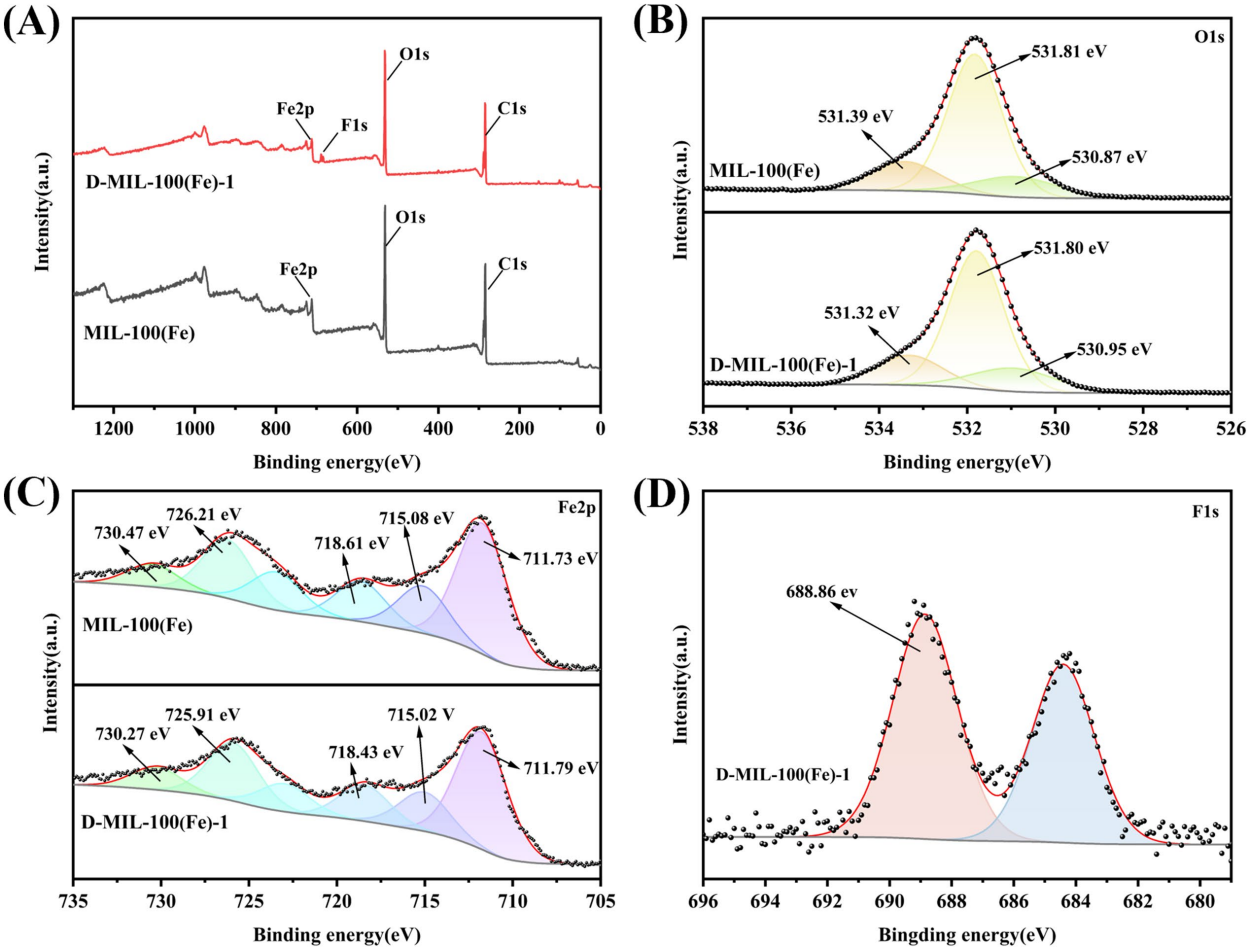
XPS spectra: **(A)** MIL-100(Fe) and D-MIL-100(Fe)-1; **(B)** O1s spectra; **(C)** Fe2p spectra; **(D)** F1s spectra.

XPS analysis was performed to confirm the successful incorporation of TFA into the D-MIL-100(Fe)-x. As shown in Figure 2A and Supplementary Figure S2, the C1s spectra exhibited binding energy peaks at 284.80 eV and 288.76 eV, corresponding to the C-C bond in the benzene ring and the O-C=O bond in the COOH group of MIL-100(Fe) and D-MIL-100(Fe), respectively. The peak near 286.55 eV was attributed to the Fe-O-C bond (39). The O1s spectra (Figures 2A,B) showed a peak at 530.95 eV associated with Fe^3+^-O bonds, a peak at 531.80 eV corresponding to Fe-O-C bonds, and a peak around 533.39 eV originating from the O-C=O group in the –COOH of H₃BTC (40). The Fe2p spectra (Figure 2C) showed two primary peaks at 711.73 eV (2p₃/₂) and 726.21 eV (2p₁/₂), indicating the presence of Fe^3+^ in both MIL-100(Fe) and D-MIL-100(Fe) (41). As shown in Figure 2C, the binding energies at 718.61 eV, 726.21 eV, and 730.47 eV in MIL-100(Fe) shifted slightly to 718.43 eV, 725.91 eV, and 730.27 eV in D-MIL-100(Fe)-1, respectively. This minor shift was likely due to coordination interactions between TFA and Fe^3+^ (42). Additionally, the appearance of a new characteristic peak at 688.86 eV in D-MIL-100(Fe)-1, which is assigned to the C–F bond, thereby demonstrates the incorporation of trifluoroacetic acid (43, 44). Collectively, these findings confirmed the successful incorporation of TFA into the framework structure of D-MIL-100(Fe).

#### 3.1.3 Nitrogen adsorption–desorption

**Figure 3:**
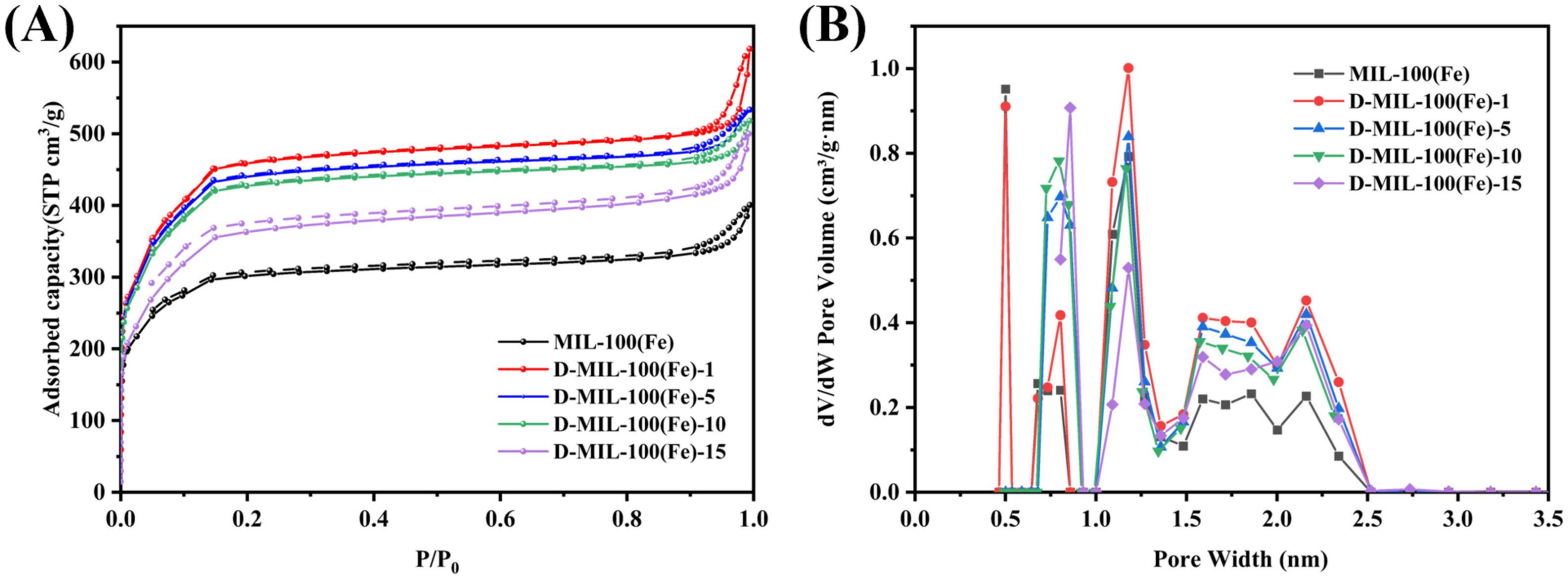
**(A)** N2 adsorption–desorption isotherms; **(B)** Pore size distribution.

The effect of TFA introduction on the loading capacity of D-MIL-100(Fe)-x was evaluated using nitrogen adsorption–desorption analysis. As shown in Figure 3A, the isotherms of D-MIL-100(Fe)-x and MIL-100(Fe) exhibited typical Type I and Type IV characteristics, indicating the coexistence of micropores and mesopores (45). The corresponding pore size distribution (Figure 3B) showed pores ranging from 0.5 to 2.5 nm. When compared with MIL-100(Fe), TFA incorporation significantly altered the pore structure by increasing the fraction of pores within 1 to 2.5 nm, aligning the pore dimensions more closely with the molecular size of eugenol (9.724 Å), the main constituent of CEO (Supplementary Figure S3). These results suggest that the enhanced CEO loading capacity of D-MIL-100(Fe)-x is driven by the optimized compatibility between the pore size and the eugenol molecule. Consequently, the significantly improved loading performance can be attributed to the effective tailoring of the pore structure via TFA modulation. This structural evolution is facilitated by the dynamic nature of coordination bonds in MOFs, which allows TFA to act as a modulator during the coordination process (46). Specifically, the introduction of TFA induces the formation of ligand defects, thereby modifying the pore structure and effectively tuning the pore dimensions (47).

Supplementary Table S1 summarizes the BET surface area and porosity parameters for MIL-100(Fe) and D-MIL-100(Fe)-x. D-MIL-100(Fe)-1 exhibited the highest surface area of 1,670.11 m^2^/g, whereas further increases in TFA content decreased the surface area. Nevertheless, D-MIL-100(Fe)-5, −10, and −15 still showed higher surface areas than MIL-100(Fe) (1,147.15 m^2^/g). The trend in specific surface area variation for these materials positively correlated with their corresponding clove essential oil loading capacities. These results demonstrate that the introduction of TFA significantly increased surface area and optimized pore architecture, suggesting enhanced CEO loading efficiency. Similar observations have been reported previously. Wang et al. (48) employed TFA as a modulator to prepare defective MOF-808, and the final defective samples exhibited the highest adsorption capacity of 860.8 mg/g. Similarly, Zhang et al. (49) synthesized defective zirconium-based MOFs using TFA as a modulator, and nitrogen sorption isotherms demonstrated high surface areas, pore volumes, and suitable pore sizes.

#### 3.1.4 Crystal structure

**Figure 4:**
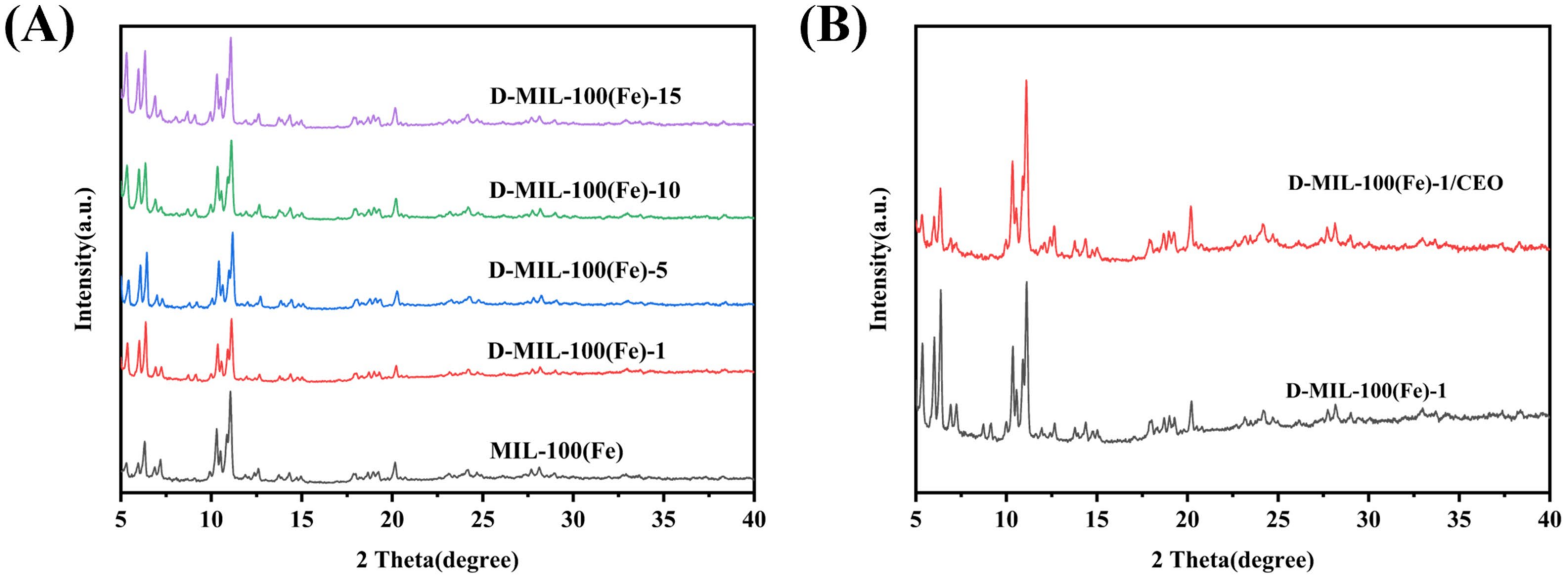
XRD spectrum. **(A)** MIL-100(Fe) and D-MIL-100(Fe)-x; **(B)** D-MIL-100(Fe)-1 and D-MIL-100(Fe)-1/CEO.

The crystal structures of MIL-100(Fe) and D-MIL-100(Fe)-1 were analyzed using XRD. As shown in Figure 4A, the diffraction peaks at 6.26°, 10.22°, and 11.00° were identified as characteristic reflections of MIL-100(Fe), confirming the successful synthesis of MIL-100(Fe), consistent with previously reported findings (50). The diffraction patterns of D-MIL-100(Fe)-x, synthesized with the addition of TFA, exhibited peaks that corresponded closely to those of MIL-100(Fe), indicating that TFA incorporation did not significantly alter the crystalline framework. Notably, the peak intensities between 5° and 6° increased with higher TFA concentrations, likely due to the participation of TFA in the nucleation process during crystal formation (51).

Following CEO loading, the XRD pattern of D-MIL-100(Fe)-1 (Figure 4B) showed that the primary diffraction peaks at 6.24°, 10.20°, and 11.02° remained unchanged, indicating that the primary diffraction crystalline framework was preserved. However, the peak intensities in the 5°–7° range decreased significantly, likely due to a small amount of CEO adhering to the surface of D-MIL-100(Fe)-1, resulting in a decrease in diffraction intensity (18). This finding was consistent with SEM results, which showed no significant morphological changes after CEO loading. Similar findings have also been reported in previous studies. Yu et al. (38) demonstrated that the crystallinity of *γ*-CD-MOF remained intact after encapsulating terpinene-4-ol, and Min et al. (52) reported decreased reflection intensities in porphyrin-MOF upon thymol incorporation while maintaining overall crystalline order. Collectively, these results confirmed the successful synthesis of D-MIL-100(Fe)-1 and the structural integrity of D-MIL-100(Fe)-1/CEO.

#### 3.1.5 FTIR spectrum

**Figure 5:**
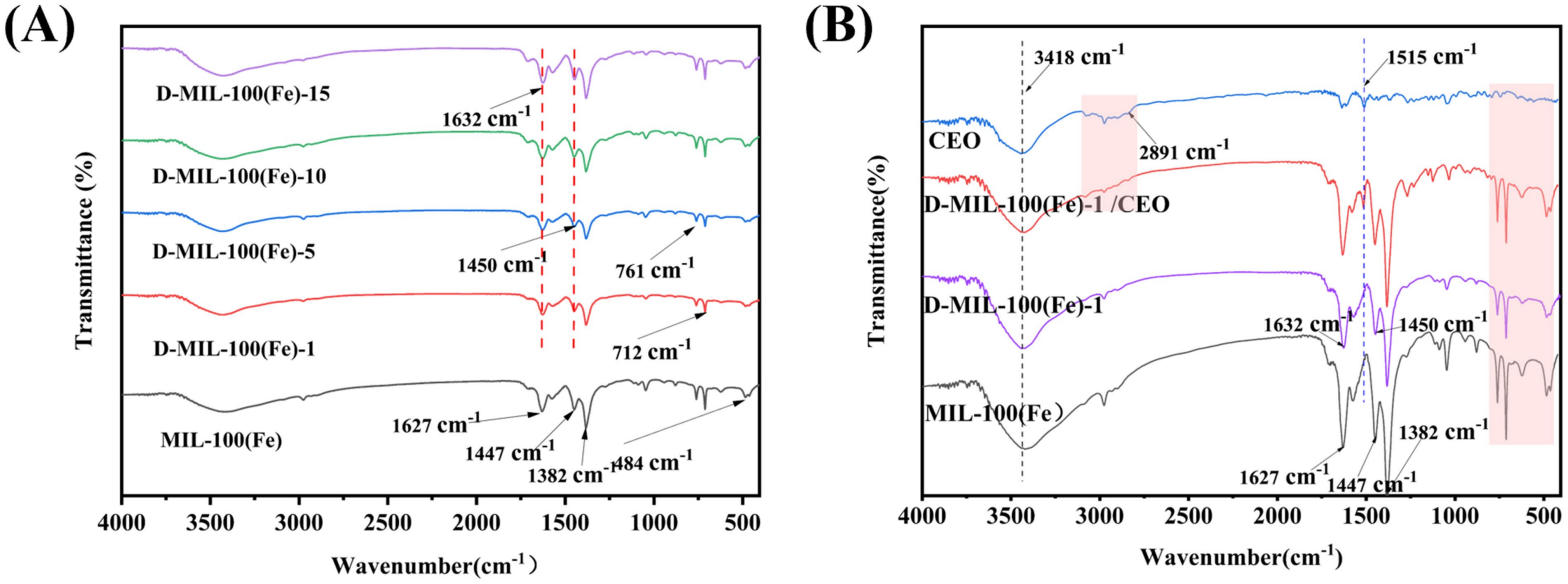
FTIR spectrum. **(A)** MIL-100(Fe) and D-MIL-100(Fe)-x; **(B)** CEO, MIL-100(Fe), D-MIL-100(Fe)-1 and D-MIL-100(Fe)-1/CEO.

The surface functional groups of CEO, MIL-100(Fe), D-MIL-100(Fe)-x, and D-MIL-100(Fe)-1/CEO were analyzed using FTIR. As shown for MIL-100(Fe) (Figure 5A), a broad band at 3,418 cm^−1^ was attributed to the -OH stretching vibration. The sharp peaks at 1,627 cm^−1^, 1,447 cm^−1^, and 1,382 cm^−1^ corresponded to the asymmetric and symmetric stretching vibrations of carboxyl groups, respectively. Additionally, the C–H bending vibrations of the benzene ring were observed at 761 cm^−1^ and 712 cm^−1^, and a peak near 484 cm^−1^ was associated with the stretching vibration of the Fe–O bond. These results were consistent with the findings of Yu et al. (53). These characteristic peaks also appeared in the FTIR spectra of D-MIL-100(Fe)-x, confirming that the fundamental structure was preserved after TFA functionalization.

Figure 5B shows that D-MIL-100(Fe)-1 loaded with CEO exhibited distinct differences compared with CEO alone, while still retaining the characteristic peaks of D-MIL-100(Fe)-1. The peaks at 1,627 cm^−1^ and 1,447 cm^−1^ in MIL-100(Fe)-1 shifted slightly to shorter wavelengths compared with those of D-MIL-100(Fe). After CEO loading, however, these peaks exhibited a blue shift to 1,632 cm^−1^ and 1,450 cm^−1^. This shift was likely due to the formation of hydrogen bonds between eugenol (the main component of CEO) and the -C=O groups of D-MIL-100(Fe)-1, causing the peaks to shift toward higher wavenumbers (54). In addition, the CEO spectrum showed an adsorption peak at 1,515 cm^−1^ corresponding to the aromatic C=C stretching vibration of eugenol, and this peak was also observed in D-MIL-100(Fe)-1/CEO.

Notably, the absorption band at 2,891 cm^−1^, which corresponds to CEO, was absent in the spectrum of D-MIL-100(Fe)-1/CEO. This observation was consistent with previous reports. Yu et al. (38) reported that the characteristic vibrational bands of terpinen-4-ol disappeared when complexed with a MOF, likely due to encapsulation within the MOF. Similarly, Shen et al. (55) observed the disappearance of the primary characteristic peak of caffeic acid in its MOF complex, indicating successful inclusion within the framework. Collectively, these results demonstrate that the nearly identical infrared spectra of D-MIL-100(Fe)-1 and D-MIL-100(Fe)-1/CEO, along with the disappearance of the CEO signature band, indicate that CEO has been effectively incorporated into D-MIL-100(Fe)-1, consistent with the SEM and XRD findings.

#### 3.1.6 Thermal properties

**Figure 6:**
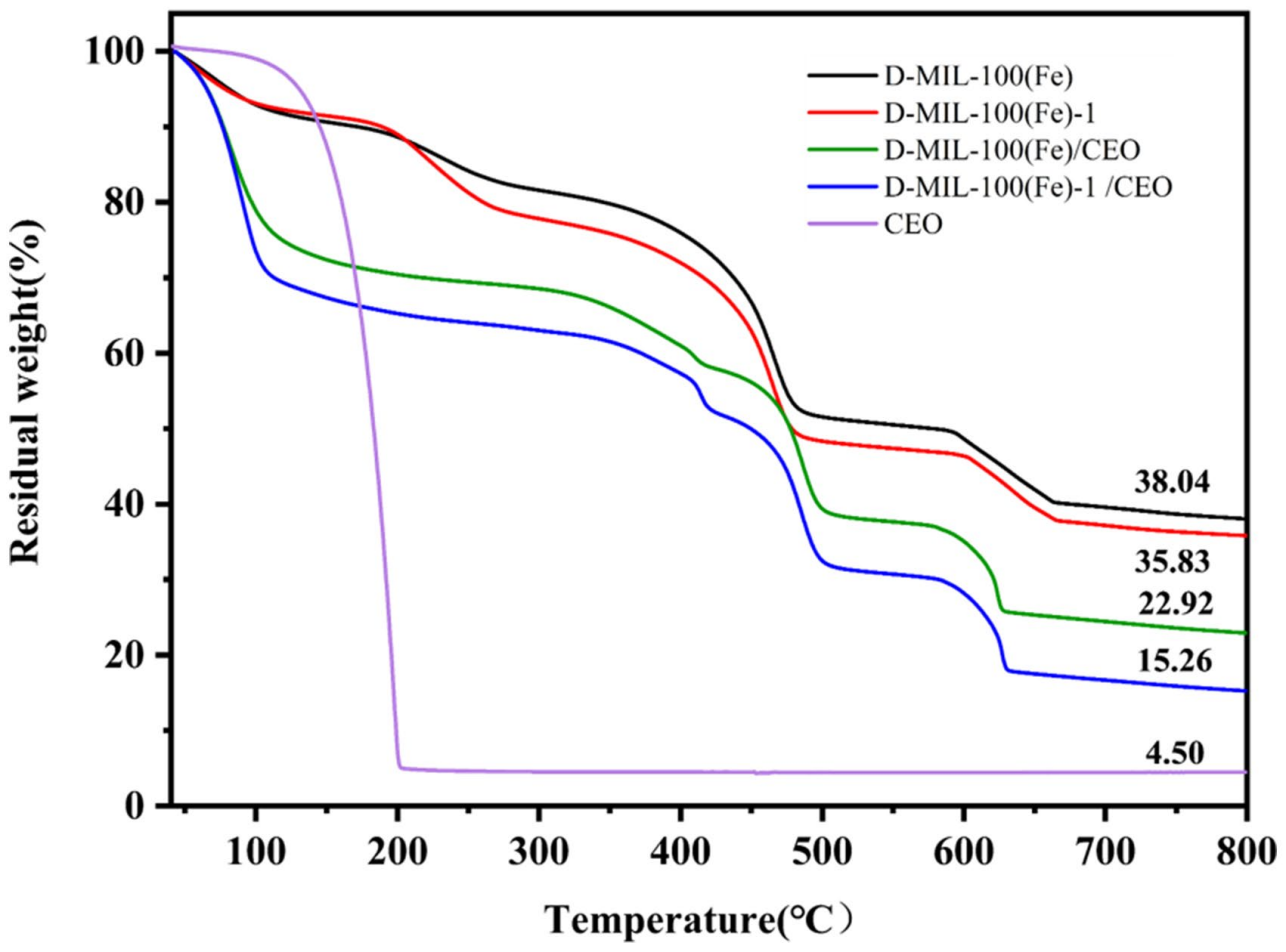
TG curves of CEO, MIL-100(Fe), D-MIL-100(Fe)-1, and D-MIL-100(Fe)-1/CEO.

The thermal stability of CEO, MIL-100(Fe), D-MIL-100(Fe)-1, and MIL-100(Fe)-1/CEO was investigated using TGA. The CEO Curve showed a mass loss of up to 95.5% between 100 and 200 °C, likely due to the volatilization of CEO (Figure 6). For MIL-100(Fe) and D-MIL-100(Fe)-1, the first weight-loss stage, occurring between 40 °C and 100 °C, was mainly due to the desorption of water and the volatilization of residual organic solvents (38). The second stage, from 100 °C to 500 °C, was attributed to the removal of anionic ligands such as OH^−^ or F^−^. The third stage, between 500 °C and 650 °C, corresponded to the decomposition of the thermally stable organic ligand H₃BTC (56). The residual weights of D-MIL-100(Fe)-1 and MIL-100(Fe) were 35.83 and 38.04%, respectively, likely due to the removal of TFA, confirming the successful integration of TFA into the framework structure of D-MIL-100(Fe)-1.

Compared with CEO, MIL-100(Fe), and D-MIL-100(Fe)-1, the weight loss of MIL-100(Fe)/CEO and D-MIL-100(Fe)-1/CEO below 105 °C was significant, demonstrating the volatilization of CEO and indicating its successful incorporation into the pores of D-MIL-100(Fe)-1. At 105 °C, the weight losses of MIL-100(Fe)/CEO and D-MIL-100(Fe)-1/CEO were 25.17 and 31.08%, respectively, suggesting a higher loading capacity for D-MIL-100(Fe)-1. Hong et al. (57) reported that MOF/CEO systems enhanced thermal stability in a concentration-dependent manner and slowed the weight loss rate of cinnamon essential oil. Similarly, Chen et al. (37) demonstrated that curcumin-loaded *γ*-CD-MOF exhibited a significantly decreased mass loss rate, indicating curcumin entrapment within the matrix and a consequent improvement in thermal stability.

### 3.2 Extraction of CEO

After the successful preparation of D-MIL-100(Fe)-x, CEO was extracted from *Syzygium aromaticum* using a salt-assisted steam distillation method (58). As shown in Supplementary Figure S4a, the yield of CEO initially increased and then decreased with increasing NaCl concentration, reaching a maximum at 3%. This phenomenon may be attributed to the ability of an appropriate NaCl concentration to regulate osmotic pressure, thereby enhancing the dissolution of CEO and improving extraction efficiency. In contrast, excessively high NaCl concentrations may inhibit the dissolution of intracellular constituents and elevate the boiling point, ultimately reducing yield (59). As shown in Supplementary Figure S4b, a liquid to solid ratio of 15 mL/g yielded the highest extraction efficiency. This ratio likely promoted sufficient contact between *Syzygium aromaticum* and steam, thereby facilitating maximum CEO yield (60). Extraction time is also a critical factor. An adequate duration ensures thorough extraction, whereas excessive time can lead to evaporation or degradation of the CEO. As shown in Supplementary Figure S4c, the yield increased from 1 to 4 h, reaching its maximum at 4 h. In summary, the optimal extraction conditions for CEO were a NaCl concentration of 3%, a liquid-to-solid ratio of 15 mL/g, and an extraction time of 4 h. These findings are consistent with a previous report (61).

### 3.3 LC(%) of CEO

**Figure 7:**
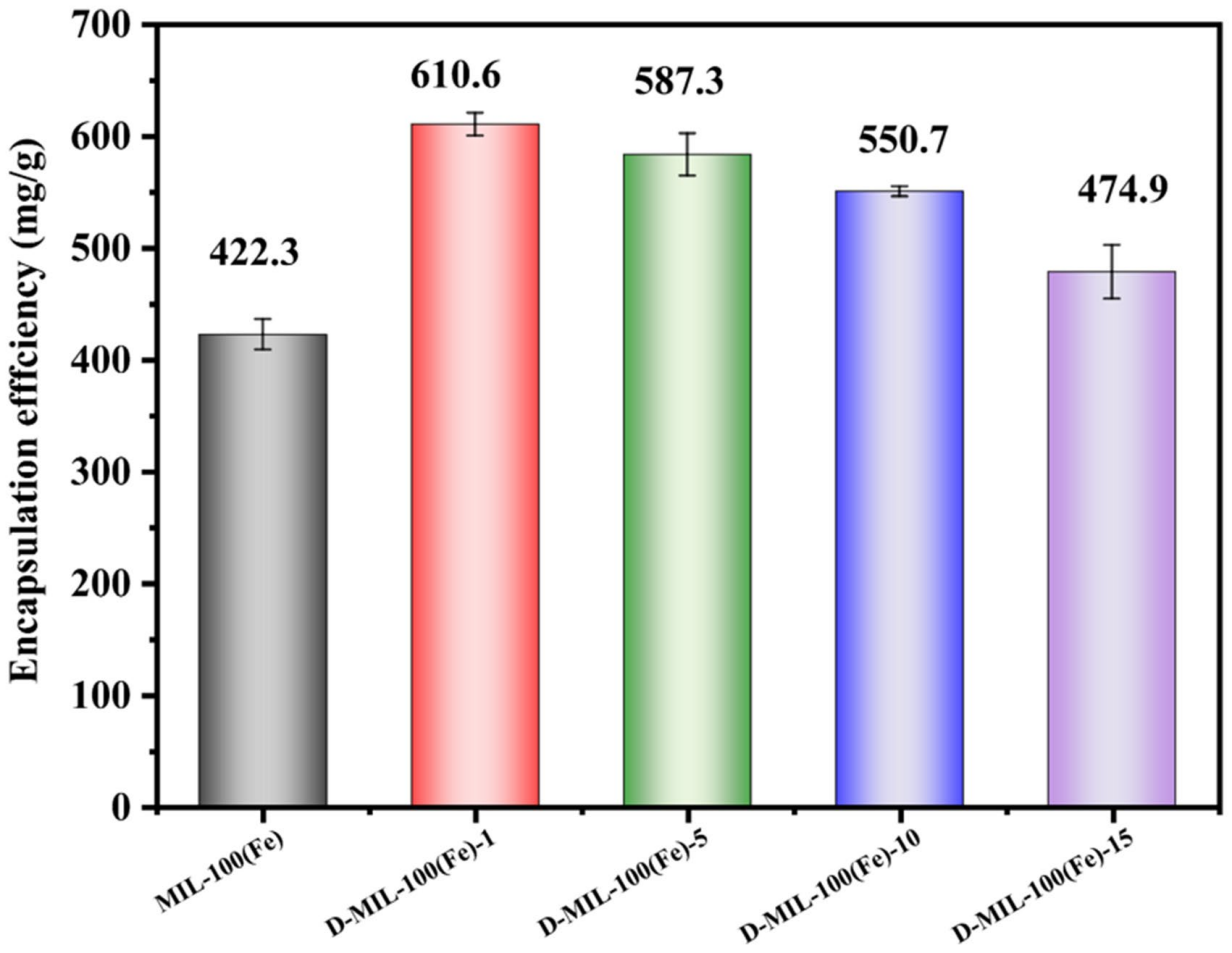
CEO loading efficiency of MIL-100(Fe) and D-MIL-100(Fe)-x.

After the successful preparation of D-MIL-100(Fe)-x/CEO, its LC(%) was investigated and compared with that of MIL-100(Fe)/CEO, as shown in Figure 7. LC of MIL-100(Fe)/CEO and D-MIL-100(Fe)-x/CEO (x = 1,5, 10, and 15) were 422.3, 610.6, 587.3, 550.7, and 474.9 mg/g, respectively. These results demonstrated that all D-MIL-100(Fe)-x samples exhibited higher LC(%) than MIL-100(Fe), with D-MIL-100(Fe)-1 achieving the highest value at 610.6 mg/g, which is 1.45 times greater than that of MIL-100(Fe). This finding is consistent with the nitrogen adsorption–desorption results.

### 3.4 *In vitro* release

**Figure 8:**
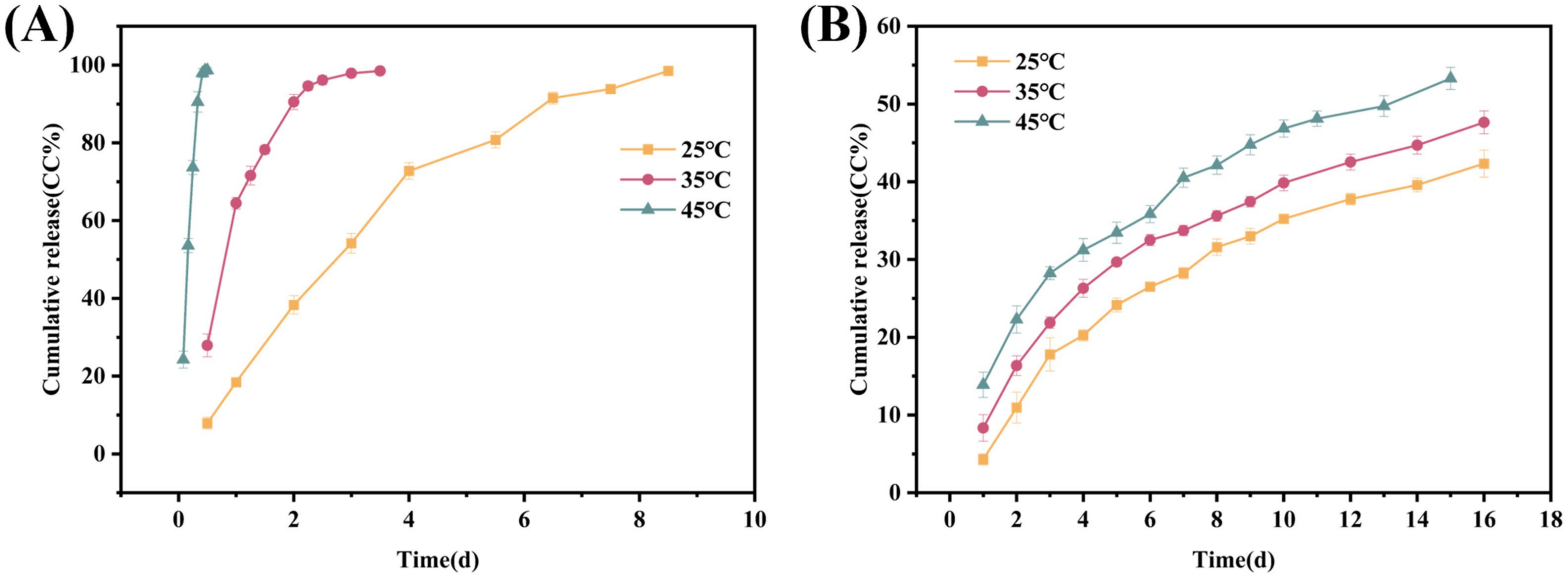
Cumulative release at 25 °C, 35 °C, and 45 °C. **(A)** CEO; **(B)** D-MIL-100(Fe)-1/CEO.

The previous results confirmed that the CEO was successfully encapsulated within D-MIL-100(Fe)-1 with an excellent loading capacity, providing a foundation for subsequent preservation and temperature-regulated delivery. The *in vitro* release profiles (Figure 8) showed a time-dependent increase in cumulative release (CR %) for both free CEO and D-MIL-100(Fe)-1/CEO. When CR % reached 40%, the release times for free CEO were 2.08, 0.63, and 0.12 d, whereas those for D-MIL-100(Fe)-1/CEO were 14.01, 9.83, and 7.01 d, respectively. These results indicated that D-MIL-100(Fe)-1/CEO provided sustained release of CEO and exhibited temperature-responsive behavior. Similarly, Yu et al. (38) reported that terpinen-4-ol loaded into *γ*-CD-MOF showed slower release than free terpinen-4-ol upon heating. Liu et al. (62) also found that terpene oils encapsulated in MOFs were released at a significantly slower rate compared with their free counterparts. The present results further demonstrated that D-MIL-100(Fe)-1 functions effectively as a carrier by reducing CEO volatilization and enhancing its thermal stability across the tested temperature range. These results were consistent with the TGA results.

To elucidate the underlying release mechanism, the data were fitted to zero-order, first-order, and Ritger–Peppas kinetic models (Supplementary Figure S5). At 25 °C, the first-order model showed the highest correlation (*R^2^* = 0.9877), indicating that CEO release was primarily determined by concentration-dependent diffusion. The Ritger–Peppas results (*R^2^* = 0.9373; *n* = 0.5952, where 0.4 < *n* < 0.89) further suggested a non-Fickian release mechanism, likely driven by a combination of matrix relaxation and diffusion processes (63, 64). Such behavior is advantageous for prolonged aroma delivery because it extends the effective service life of the entrapped CEO.

### 3.5 Antioxidant and antibacterial activity

#### 3.5.1 Antioxidant assay

Reactive oxygen species and free radicals contribute to organic spoilage through lipid peroxidation and protein denaturation (65). To determine whether encapsulation mitigates CEO deterioration, the DPPH-scavenging capacities of CEO, D-MIL-100(Fe)-1, and the composite D-MIL-100(Fe)-1/CEO were monitored over a five-day storage period (Supplementary Figure S6). At the beginning of the experiment, both CEO and D-MIL-100(Fe)-1/CEO showed the highest antioxidant activity. As storage progressed, the antioxidant activity of free CEO decreased almost linearly, reflecting the rapid volatilization of eugenol. In contrast, D-MIL-100(Fe)-1/CEO retained more than 50–70% of its initial activity throughout the same period, showing only a gradual decrease. By day 5, the remaining DPPH-scavenging activity of D-MIL-100(Fe)-1/CEO was approximately five fold higher than that of free CEO. This difference likely resulted from the rapid evaporation of free CEO, which diminished its antioxidant capacity. In contrast, the CEO encapsulated in D-MIL-100(Fe)-1 diffused slowly from the pores of D-MIL-100(Fe)-1. These results demonstrated that the antioxidant activity of D-MIL-100(Fe)-1/CEO was more sustained than that of free CEO, consistent with previous observations reported by Chen et al. (66).

The prolonged DPPH radical-scavenging behavior of D-MIL-100(Fe)-1/CEO resulted from the combined contributions of CEO and the MOF matrix. The CEO provides eugenol, which donates hydrogen atoms to peroxyl and superoxide radicals, acting as a chain-breaking antioxidant (67). Simultaneously, D-MIL-100(Fe)-1 quenched singlet oxygen, reduced superoxide radicals, and chelated pro-oxidant metal ions through its surface functional groups, including –NH₂, –OH, and –COOH (68). Collectively, these effects provide a long-acting oxygen-blocking mechanism suitable for preservation applications.

#### 3.5.2 Antibacterial assay

Bacterial colonization is the primary cause of post-harvest fruit deterioration (69). Studies indicate that the antibacterial action of EOs microdroplets is initiated by their adhesion to the bacterial surface via solid liquid triboelectrification (70–73). This initial interaction suppresses the synthesis of cell wall glucans, impedes bacterial motility and the induction of quorum sensing, and compromises the structure and function of the cytoplasmic membrane and its proteins. These primary effects culminate in cellular damage, including the leakage of intracellular components, cytoplasmic condensation, and disruption of the proton motive force (74). Consequently, CEO demonstrates broad spectrum inhibitory activity against a wide range of bacteria (75, 76). In this study, the antibacterial activity of D-MIL-100(Fe)-1/CEO was investigated against *Staphylococcus aureus*, *Escherichia coli*, and *Bacillus subtilis* over a 5 day storage period (Supplementary Figure S7). At the beginning of the experiment, both free CEO and D-MIL-100(Fe)-1/CEO exhibited high inhibitory effects against all three indicator strains. As storage progressed, the antibacterial activity of free CEO decreased sharply, whereas that of the D-MIL-100(Fe)-1/CEO composite decreased gradually. After 120 h, the inhibition zones produced by the composite remained 2.8–3.2 times larger than those of free CEO, likely due to the slow-release behavior of the encapsulated CEO, which maintained local concentrations above the minimum inhibitory threshold for an extended period (77).

Ampicillin (50 μg/disc) was used as a positive control and exhibited Minimum inhibitory concentration (MIC) equivalent of 19.65 mM (*Escherichia coli*), 15.12 mM (*Bacillus subtilis*), and 14.32 mM (*Staphylococcus aureus*). Although the composite does not match the potency of the antibiotic, D-MIL-100(Fe)-1/CEO offers the advantage of prolonged, plant-derived antimicrobial activity without reliance on chemical agents. Similar observations were reported by Caamaño et al. (78), who found that a carvacrol-loaded MOF exhibited extended antibacterial effects against *Escherichia coli* and *Listeria innocua*. Collectively, these results demonstrated that D-MIL-100(Fe)-1/CEO functioned as a slow-release, plant-derived antimicrobial agent suitable for inhibiting microbial spoilage during preservation.

### 3.6 Preservation assay

The D-MIL-100(Fe)-1/CEO composite combined high LC(%) with diffusion-controlled release, resulting in prolonged antioxidant and antibacterial activity that can contribute to practical fruit preservation. As shown in Supplementary Figure S8, all samples appeared similar across all groups on day 0. By day 5, the untreated control fruit exhibited evident deterioration, including browning of the epidermis and underlying flesh, together with visible signs of spoilage. In contrast, peaches treated with D-MIL-100(Fe)-1/CEO remained plump and green-orange, exhibiting a preservative effect comparable to that of the refrigerated group. By the final day, the untreated peaches were entirely covered by a dense mycelial layer and had lost all marketability. In contrast, the D-MIL-100(Fe)-1/CEO treated peaches displayed only slight localized discoloration and no skin rupture. This outcome was likely attributable to the continuous release of the CEO, which suppressed bacterial proliferation and oxidative browning, thereby extending shelf life. A similar trend was observed in strawberries treated with citral microcapsules, supporting the value of MOF-based delivery systems for room-temperature preservation (66).

**Figure 9:**
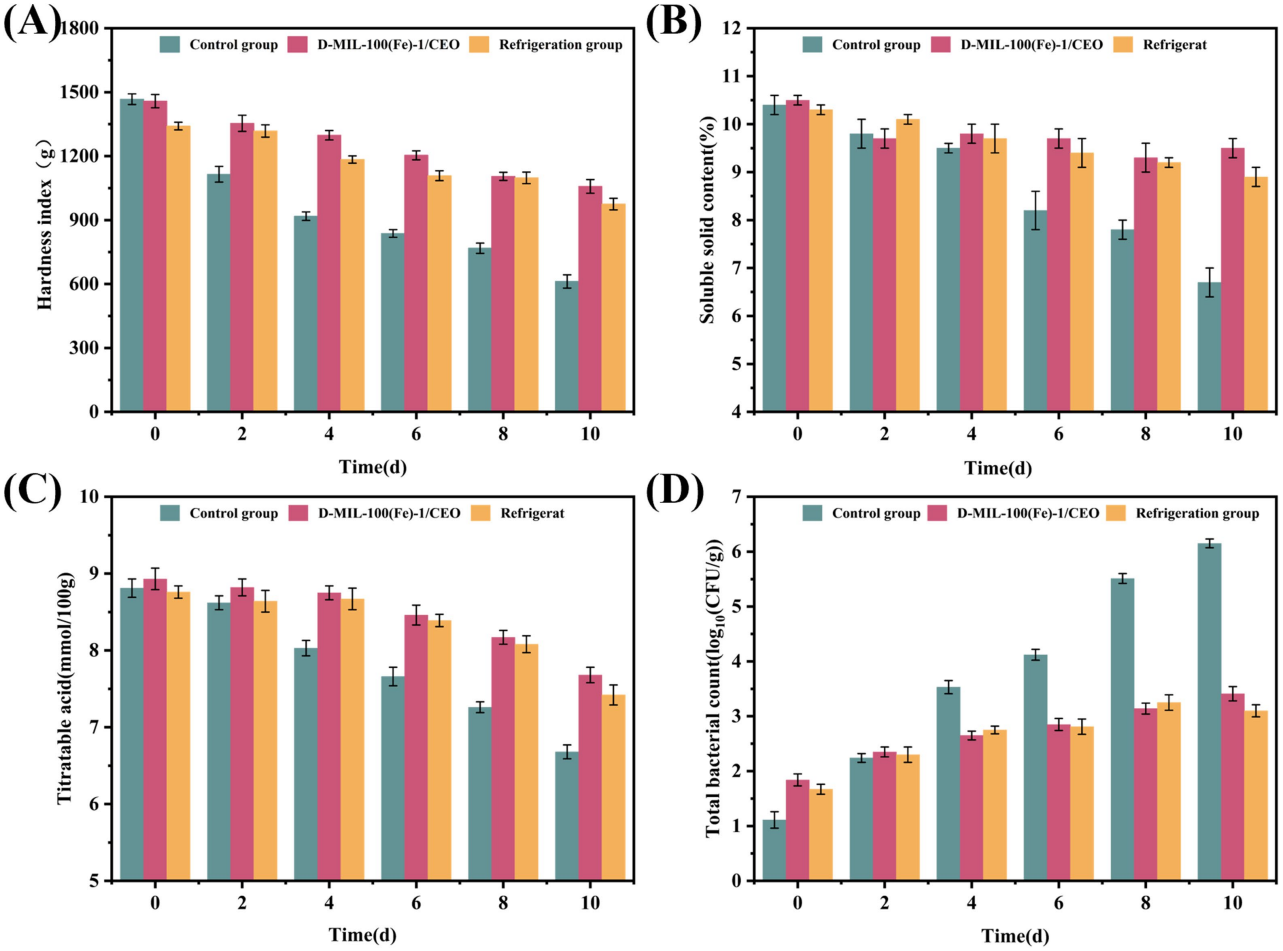
Preservation assay of D-MIL-100(Fe)-1/CEO. **(A)** hardness; **(B)** SSC; **(C)** TA; **(D)** TBC.

Cell-wall softening is primarily governed by pectin, which is demethylated to form pectic acid, resulting in tissue tenderization(66). Over the 10 day storage period, the firmness of the untreated control peaches decreased significantly, decreasing by nearly 60% from 1,467 to 612 g/cm (Figure 9A), resulting in visual deterioration. In contrast, peaches treated with D-MIL-100(Fe)-1/CEO, as well as those stored at 4 °C, retained approximately 75% of their initial firmness, indicating that the sustained release of the CEO helped maintain cell-wall integrity.

SSC indicates the balance between starch-to-sugar conversion and respiratory losses (79). In the untreated control group, SSC decreased from 10.4 to 6.7%, whereas peaches treated with D-MIL-100(Fe)-1/CEO declined by only about 1% (Figure 9B), closely resembling the behavior observed in the refrigerated group. This preservation of SSC indicated that the continuous release of CEO suppressed the respiration rate, thereby preserving the sweetness of the peaches.

As shown in Figure 9C, TA decreased by 2.13 mmol/L in the untreated peaches but by only 1.25 mmol/L in the D-MIL-100(Fe)-1/CEO-treated peaches, indicating that CEO slowed acid catabolism and limited the associated microbial proliferation (80).

Microbial safety was assessed by measuring TBC. In the untreated control, TBC exceeded 3.5 Log₁₀ CFU/g within 4 days and surpassed the 6 Log₁₀ CFU/g threshold by day 10, rendering the peach unfit for consumption. In contrast, the D-MIL-100(Fe)-1/CEO-treated peaches maintained a final bacterial load below 6 Log₁₀ CFU/g, increasing only from 2.96 to 5.95 Log₁₀ CFU/g (Figure 9D), likely due to the sustained vapor-phase antibacterial activity of CEO. Hong et al. (57) previously reported a comparable 99.9% reduction in microbial growth for Gel/Pull/CEO@MOF films applied to fresh meat.

Overall, the texture, chemical parameters, and microbiological profiles indicated that D-MIL-100(Fe)-1/CEO provided preservation efficacy at room temperature comparable to refrigeration, D-MIL-100(Fe)-1 is synthesized via a hydrothermal method, featuring a simple synthesis process and low production cost. It achieves an exceptionally high loading capacity for CEO through simple immersion. This effectively enables controlled release of natural essential oils, prolonging their efficacy and enhancing the preservation effect of essential oils on peaches, offering an energy-efficient strategy for extending the shelf life of peaches.

## 4 Conclusion

In this study, trifluoroacetic acid was used as a modulator to synthesize defective D-MIL-100(Fe)-x, which was then used as a loading material for CEO. Screening results showed that D-MIL-100(Fe)-1 had the highest CEO loading capacity, reaching 610.6 mg/g, representing a 1.45-fold increase compared with conventional MIL-100(Fe) (422.3 mg/g). The incorporation of TFA significantly increased the specific surface area and modified the pore structure of D-MIL-100(Fe)-1. These structural changes likely contributed to its enhanced loading capacity. The CEO loaded onto D-MIL-100(Fe)-1 released 42.3% over 16 days at 25 °C, indicating a significant sustained release effect. When applied as a preservative for peaches, D-MIL-100(Fe)-1/CEO significantly reduced softening and browning, minimized losses in soluble solids and titratable acidity, and inhibited bacterial proliferation throughout the 10 day storage period. Consequently, the composite provided comprehensive protection of peach quality, flavor, and texture, extended shelf life, and alleviated challenges related to transportation. Overall, a novel defective MIL-100(Fe)-x serves as an efficient carrier for CEO and offers a safe and biocompatible approach for fruit and vegetable preservation, supporting advancements in quality and safety within the food industry.

## Data Availability

The original contributions presented in the study are included in the article/Supplementary material, further inquiries can be directed to the corresponding authors.
