# Supplementary material for: Temperature-regulated defective MIL-100(Fe) for clove essential oil loading as an effective natural preservative for peaches
Source: Front Nutr. 2026 Apr 16;13:1755325. doi: 10.3389/fnut.2026.1755325 (PMC13131099; doi:10.3389/fnut.2026.1755325)
Supplement: Supplementary file 1 [file Table_1.docx]

**Appendix A. Supplementary data**

**Temperature-regulated defective MIL-100(Fe) for clove essential oil loading as an effective natural preservative for peaches**

Naiding Wang ^1♀^, Jun Zhang^3♀^, Rongpeng Li^3♀^, Yanqing Duan^1^, Ruizhi Zhu ^2^, Lei Yang ^2^, Haotian Yang ^1^, Yuandong Li ^1^, Juxing Jiang ^1^, Yanqun Xu ^2*^, Zhigang Tai ^3^^[[1]](#footnote-1)^

（1.Yunnan Tobacco Industrial Hi-tech Material Co., Ltd, Kunming 650106; 2. R＆D Central of China Tobacco Yunnan Industrial Co., Ltd, Kunming 650106; 3. Faculty of Science and Life Technology, Kunming University of Science and Technology, Kunming 650500, China）


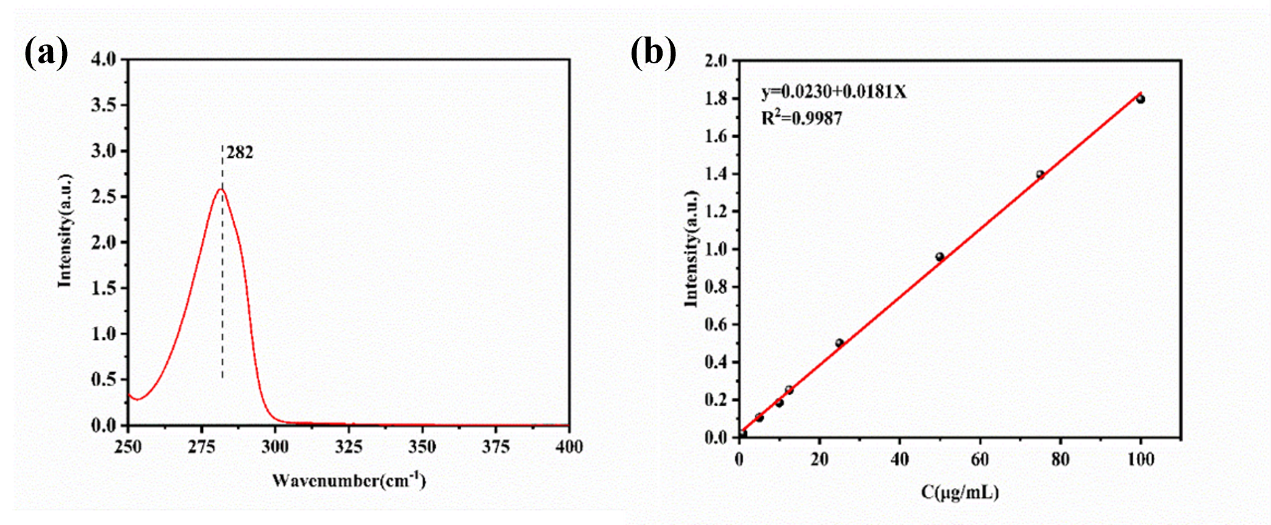


**Fig.S1. (a)Ultraviolet absorption of CEO;(b) Standard curve for CEO.**


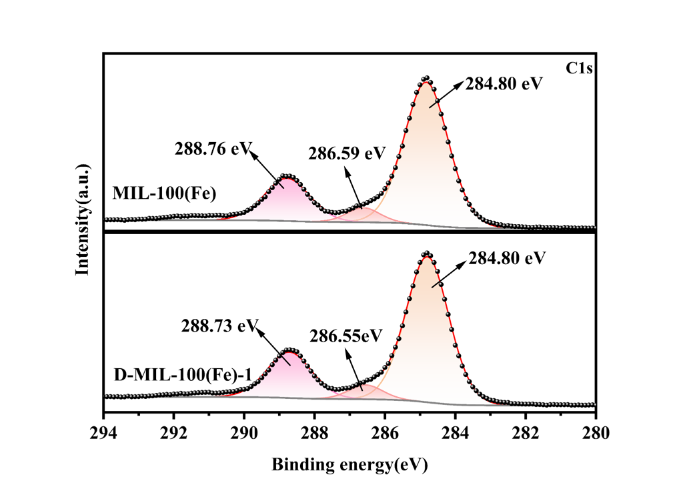


**Fig.S2. XPS of C1s spectra (MIL-100(Fe) and D-MIL-100(Fe)-1 )**


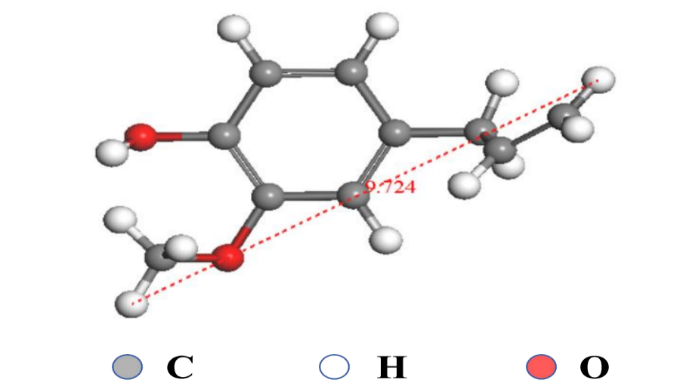


**Fig.S3. Molecular structure and size of eugenol of CEO.**


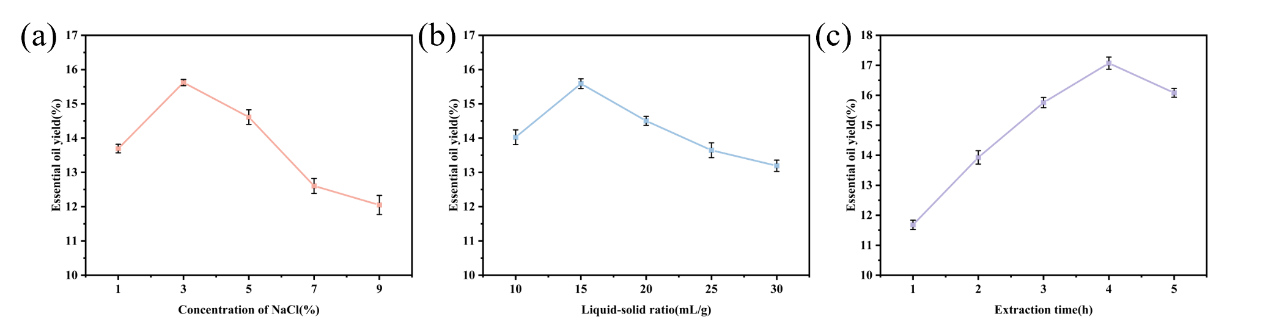


**Fig.S4. (a) Effect of** **sodium chloride; (b) Effect of liquid–solid ratio; (c) Effect of extraction time.**


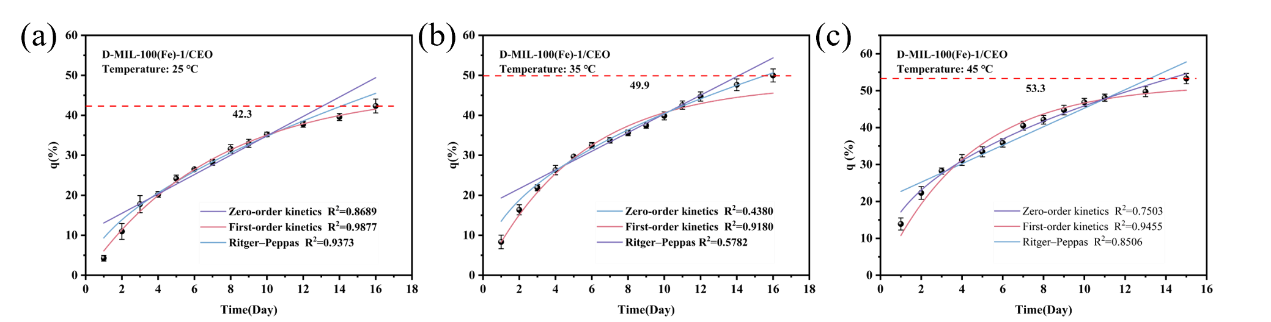
 **Fig.S5. (a) Zero-order, (b)First-order, a (c)Ritger–Peppas kinetics models.**


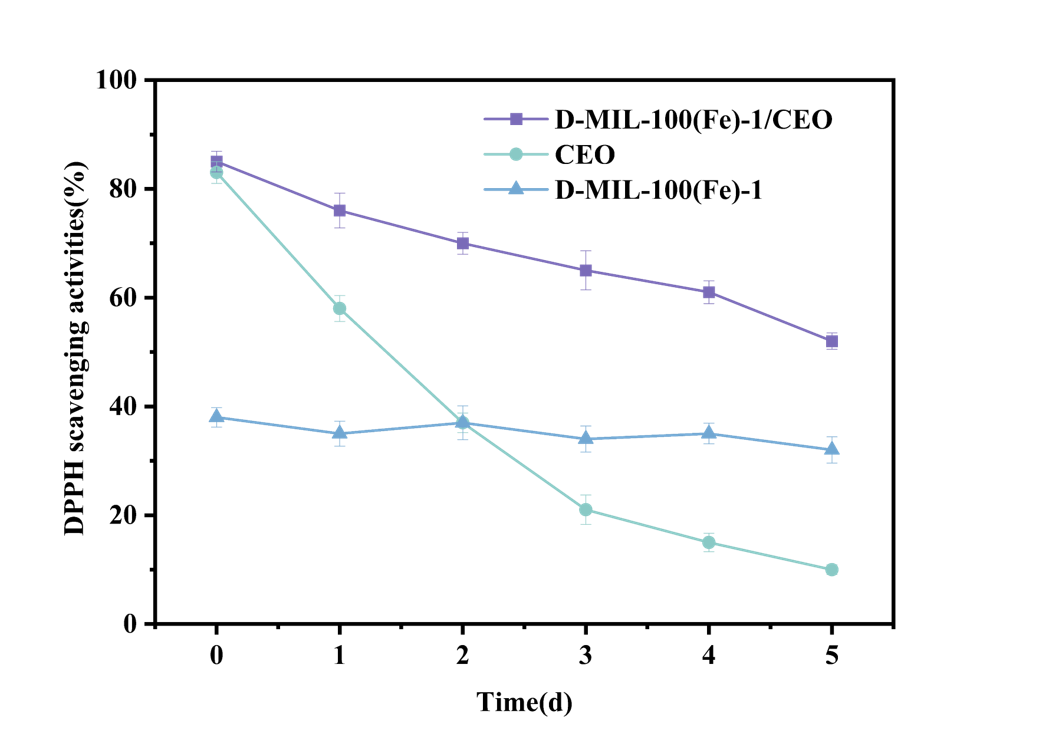


**Fig.S6. DPPH scavenging activity of CEO,** **D-MIL-(Fe)-1, and D-MIL-(Fe)-1/CEO**


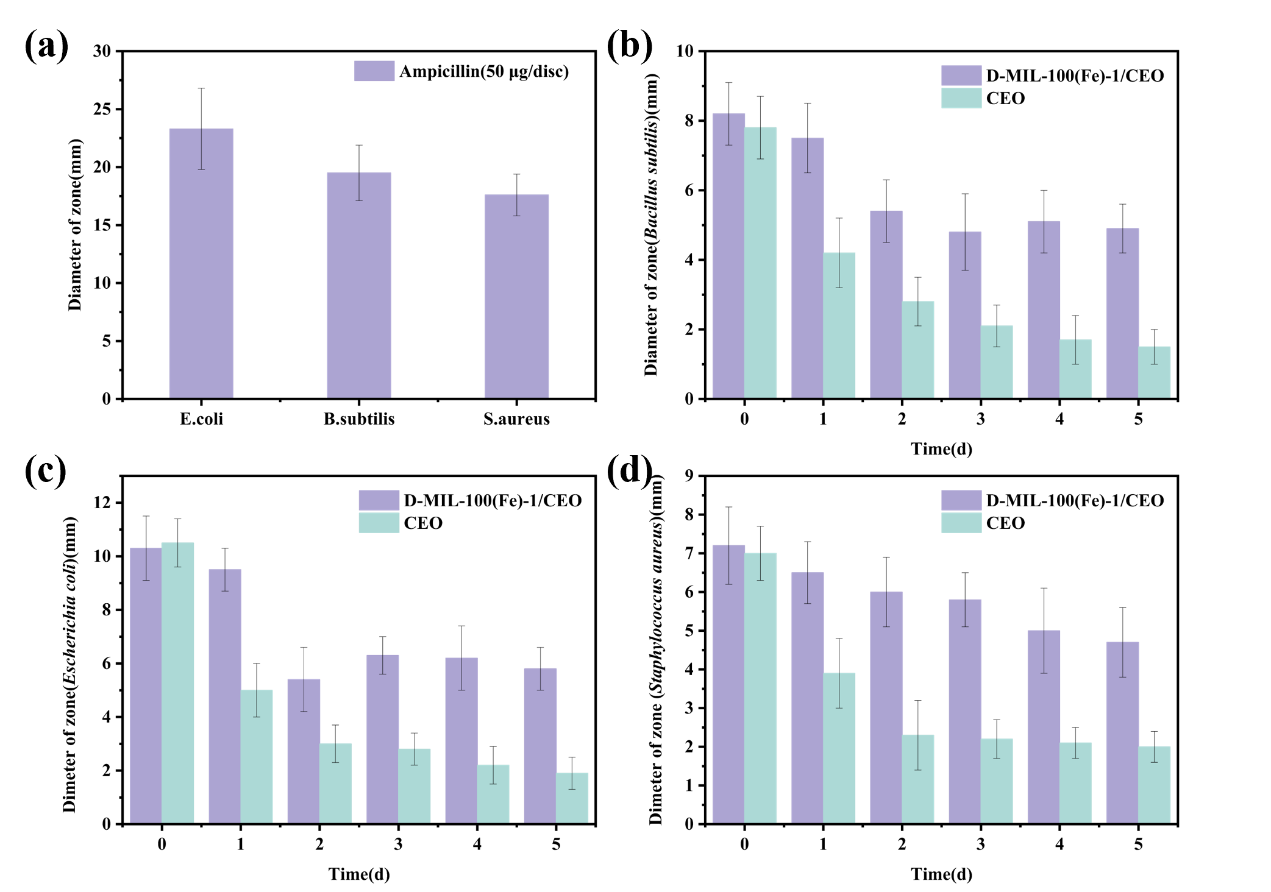


**Fig. S7. Antibacterial activities of CEO and D-MIL-100(Fe)-1/CEO activity over 5 days**


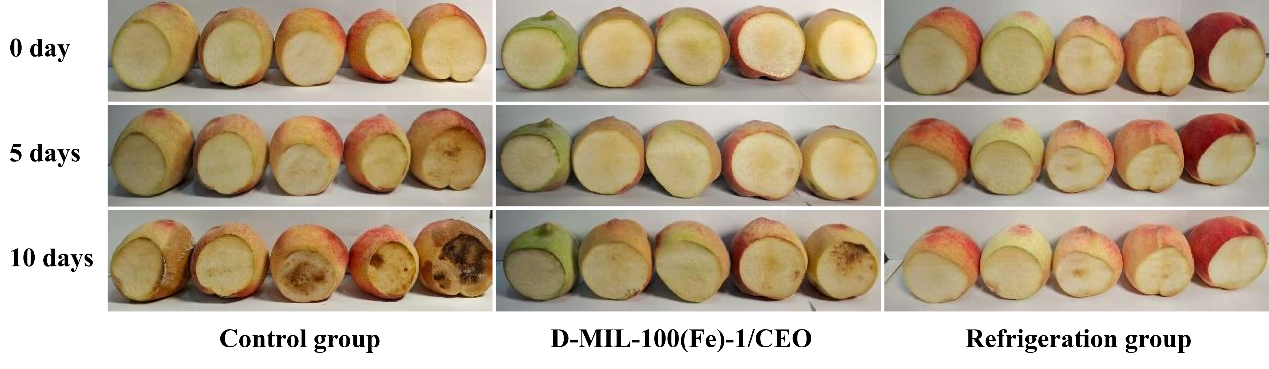


**Fig.S8. Changes in the appearance of peaches stored for 10 days under different conditions.**

**Table S1. BET surface area and porosity parameters for MIL-100(Fe) and D-MIL-100(Fe)-x**

| Materials | BET Surface Area（m^2^/g) | t-Plot micropore volume（cm^3^/g) |
| --- | --- | --- |
| MIL-100(Fe） | 1147.15 | 0.44 |
| D-MIL-100(Fe)1 | 1670.11 | 0.65 |
| D-MIL-100(Fe)-5 | 1640.16 | 0.63 |
| D-MIL-100(Fe)-10 | 1560.62 | 0.59 |
| D-MIL-100(Fe)-15 | 1422.00 | 0.51 |

**Table S2. Graphical fitting and the correlation coefficients of Zero-order, First-order, and Ritger–Peppas kinetics models**

| Kinetic model | | | 25 ℃ | 35 ℃ | 45 ℃ |
| --- | --- | --- | --- | --- | --- |
| Zero-order kinetics | $\text{q}\text{ }\text{=}{\text{ }\text{k}}_{\text{1}}\text{t}\text{ + }\text{c}_{\text{1}}$ | *k_1_* | 0.2042 | 0.0383 | 0.3642 |
|  |  | *C_1_* | 0.1028 | 0.8499 | 1.3484 |
|  |  | *R^2^* | 0.8689 | 0.4380 | 0.7503 |
| First-order kinetics | $\text{q}\text{ }\text{= 1-}\text{e}^{\text{-}\text{k}_{\text{2}}\text{t}}$ | *k_2_* | 0.2372 | 1.2417 | 0.4525 |
|  |  | *R^2^* | 0.9877 | 0.9180 | 0.9455 |
| Ritger-Peppas | $\text{q}\text{ }\text{=}{\text{ }\text{k}}_{\text{3}}\text{t}^{\text{n}}$ | *k_3_* | 0.2912 | 0.8440 | 1.4479 |
|  |  | *n* | 0.5952 | 0.1233 | 0.4962 |
|  |  | *R^2^* | 0.9373 | 0.5782 | 0.8506 |

1. ^♀^Naiding Wang, Jun Zhang, and Rongpeng Li contributed equally to this work and should be considered co-first authors.

   Corresponding Authors: Yanqun Xu (E-mail: xuyq@ynzy-tobacco.com); Zhigang Tai (E-mail: taizg@kust.edu.cn) [↑](#footnote-ref-1)
